# ACE2 Shedding and the Role in COVID-19

**DOI:** 10.3389/fcimb.2021.789180

**Published:** 2022-01-14

**Authors:** Jieqiong Wang, Huiying Zhao, Youzhong An

**Affiliations:** Department of Critical Care Medicine, Peking University People’s Hospital, Beijing, China

**Keywords:** soluble angiotensin converting enzyme 2, severe acute respiratory syndrome coronavirus 2, treatment, angiotensin converting enzyme 2, COVID-19

## Abstract

Angiotensin converting enzyme 2 (ACE2), a transmembrane glycoprotein, is an important part of the renin-angiotensin system (RAS). In the COVID-19 epidemic, it was found to be the receptor of severe acute respiratory syndrome coronavirus 2 (SARS-COV-2). ACE2 maintains homeostasis by inhibiting the Ang II-AT1R axis and activating the Ang I (1-7)-MasR axis, protecting against lung, heart and kidney injury. In addition, ACE2 helps transport amino acids across the membrane. ACE2 sheds from the membrane, producing soluble ACE2 (sACE2). Previous studies have pointed out that sACE2 plays a role in the pathology of the disease, but the underlying mechanism is not yet clear. Recent studies have confirmed that sACE2 can also act as the receptor of SARS-COV-2, mediating viral entry into the cell and then spreading to the infective area. Elevated concentrations of sACE2 are more related to disease. Recombinant human ACE2, an exogenous soluble ACE2, can be used to supplement endogenous ACE2. It may represent a potent COVID-19 treatment in the future. However, the specific administration concentration needs to be further investigated.

## Introduction

Coronavirus disease 2019 (COVID-19), caused by a novel strain of severe acute respiratory syndrome coronavirus 2 (SARS-COV-2), has become a worldwide pandemic, endangering the health and economy of humans. Angiotensin converting enzyme 2 (ACE2) is the cell membrane receptor of SARS-COV-2, mediating viral entry into cells (Hoffmann et al., 2020). ACE2 has already been identified as a SARS-COV receptor, while the affinity of ACE2 binding to SARS-COV-2 is 10~20-fold higher than that of ACE2 binding with SARS-COV (Wrapp et al., 2020). Although ACE2 anchors onto the cell surface, it is not stable, and can shed from the membrane, which is referred to as ACE2 shedding (Lambert et al., 2005). ACE2 shedding produces soluble ACE2 (sACE2), resulting in loss of the membrane-bound form. Whether ACE2 shedding and increasing sACE2 are physiological or pathological has not been clearly elucidated. Recently, sACE2 has been found to facilitate SARS-COV-2 infection in cells (Karthika et al., 2021; Yeung et al., 2021). On the other hand, clinical-grade recombinant human ACE2 (rhACE2), a type of exogenous soluble form of ACE2, binds to SARS-COV-2 in ﻿engineered human tissues and inhibits virus infection (Monteil et al., 2020). Therefore, the role sACE2 plays in COVID-19 warrants further study.

**Figure 1:**
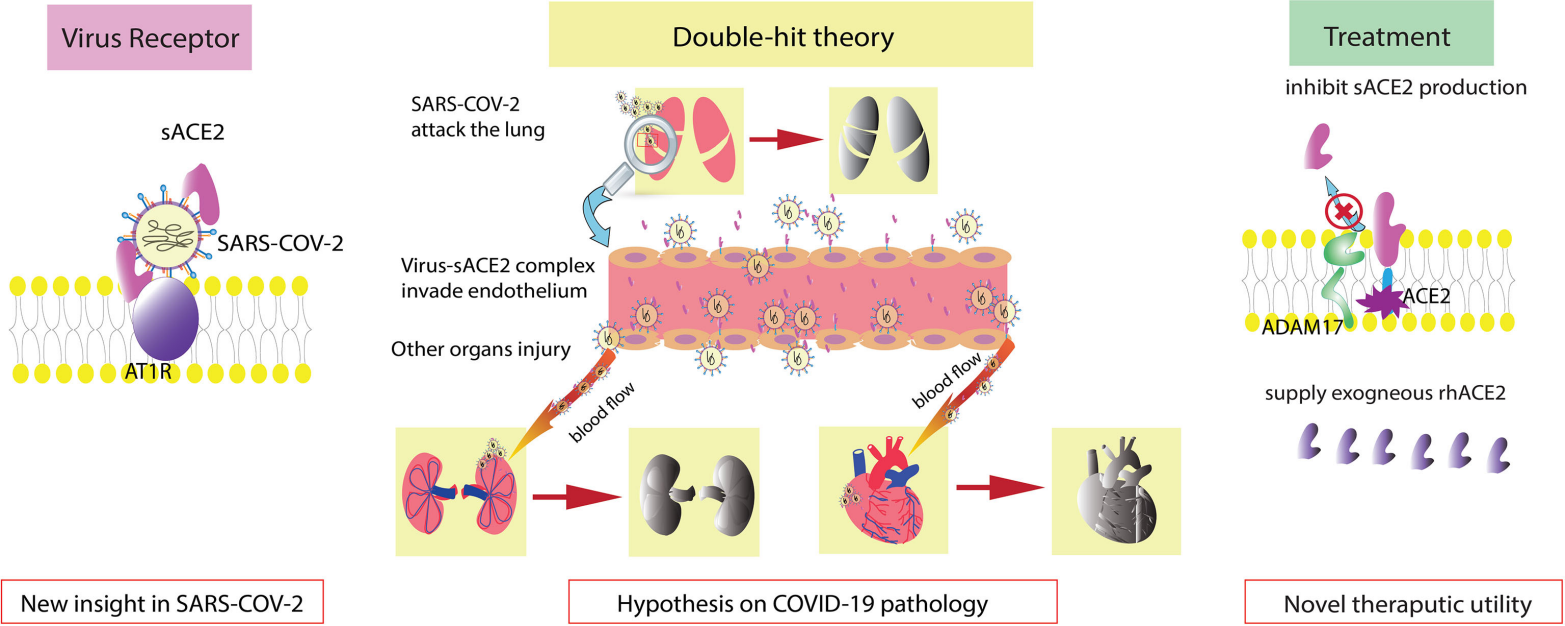
Graphical abstract. sACE2 is also a receptor of SARS-COV-2. Double-hit theory: endothelial injury exacerbates the severity of COVID-19. Regulation of sACE2 production and supplementation with exogenous rhACE2 are new therapeutic options.

In this narrative review, we focus on the generative mechanism of sACE2 and sACE2 in COVID-19 and the therapeutic use of rhACE2 in COVID-19 (**Figure 1**).

## The Structure and Function of ACE2

ACE2, discovered in 2000 as the homolog of ACE, is a type I transmembrane glycoprotein that resides on the cell surface (Tipnis et al., 2000). ACE2 is broadly distributed throughout the human body. It is expressed in the kidney, testis, intestine, lung, retina, cardiovascular system, adipose tissue and central nervous system (Hamming et al., 2004; Gheblawi et al., 2020). The human ACE2 gene maps to chromosome Xp22 and contains 18 exons (Tipnis et al., 2000). The ACE2 protein, which has a full length of 805 amino acids, exhibits an extracellular N-terminal claw-like protease domain (PD) and a C-terminal collectrin-like domain (CLD) with a cytosolic tail (Zhang et al., 2001; Gheblawi et al., 2020). The PD of the N-terminus can bind to the receptor binding domain (RBD) of Spike protein both SARS-COV and SARS-COV-2, forming the PD-RBD complex and facilitating virus entry (Li et al., 2003; Hoffmann et al., 2020). The affinity of ACE2 binding to SARS-COV-2 is 10~20-fold higher than that of SARS-COV, which may explain the severity of COVID-19 (Wrapp et al., 2020). Distinct from the virus binding site, the HEXXH zinc binding metalloprotease motif in the N-terminus exerts carboxypeptidase function, which converts angiotensin I (Ang I) to Ang 1-9 or Ang II to Ang I 1-7 (Vickers et al., 2002). In addition, ACE2 cuts the C-terminal residue from three other vasoactive peptides, neurotensin, kinetensin, and des-Arg bradykinin (Vickers et al., 2002). In contrast, ACE converts Ang I to Ang II and cleaves bradykinin. The counterbalance of ACE-Ang II-angiotensin II type 1 receptor (AT1R) and ACE2-Ang I (1-7)-mitochondrial assembly receptor (MasR) plays an important role in RAS. Increasing and activating the ACE2-Ang I (1-7)-MasR axis reduces cytokine release and protects against organ injury in many human diseases, including cardiovascular disease, obesity, chronic kidney disease, liver diseases and lung injury (Rodrigues Prestes et al., 2017). On the other hand, the intracellular CLD of ACE2 participates in amino acid transport by regulating the epithelial neutral amino acid transporter B0AT1 in the small intestine (Camargo et al., 2009).

## sACE2 Is Generated From ACE2 Shedding

Additionally, ADAM17, a disintegrin and metallopeptidase domain 17 (ADAM17)/tumor necrosis factor α-converting enzyme (TACE), cleaves ACE2 from the cell surface, generating a soluble, enzymatically active ectodomain form of the enzyme sACE2. ADAM17-induced ACE2 shedding is activated by phorbol ester (PMA) (Lambert et al., 2005). Lambert et al. confirmed the presence of ectodomain shedding of heterologously expressed ACE2 in HEK293 cells and endogenously expressed ACE2 in Huh7 cells (Lambert et al., 2005). Rice et al. first detected circulating ACE2 in healthy individuals, although the concentration of ACE2 was far lower than that of ACE (Rice et al., 2006). Subsequently, researchers discovered that calmodulin’s interaction with the cytoplasmic tail of ACE2 inhibited its shedding, which was independent of PMA-mediated shedding (Lambert et al., 2008; Lai et al., 2009). A study on human epithelial cells indicated that ADAM17 exerted its sheddase function *via* the ectodomain of ACE2 (Jia et al., 2009). It seems that calmodulin and ADAM17 affect ACE2 proteolytic cleavage through two dependent mechanisms. However, Mou et al. found that dissociation of calmodulin from semaphorin 4D (Sema4D) in platelets was sufficient to trigger ADAM17-dependent Sema4D cleavage (Mou et al., 2013). Whether calmodulin and ADAM-17 apply a similar method to regulate ACE2 shedding or other possible crosstalk between them needs to be further investigated.

On the other hand, transmembrane protease serine 2 (TMPRSS2) competes with ADAM17 in ACE2 cleavage (Shulla et al., 2011; Heurich et al., 2014). In contrast, TMPRSS2 requires arginine and lysine residues within ACE2 amino acids 697 to 716 for receptor cleavage, while ADAM17 acts within sites 652 to 659 (Heurich et al., 2014). In addition, TMPRSS2 primes the virus spike protein of both SARS-COV and SARS-COV-2, activating the S protein for membrane fusion (Heurich et al., 2014; Hoffmann et al., 2020). An inhibitor of TMPRSS2 might represent a new therapeutic target in COVID-19.

**Figure 2:**
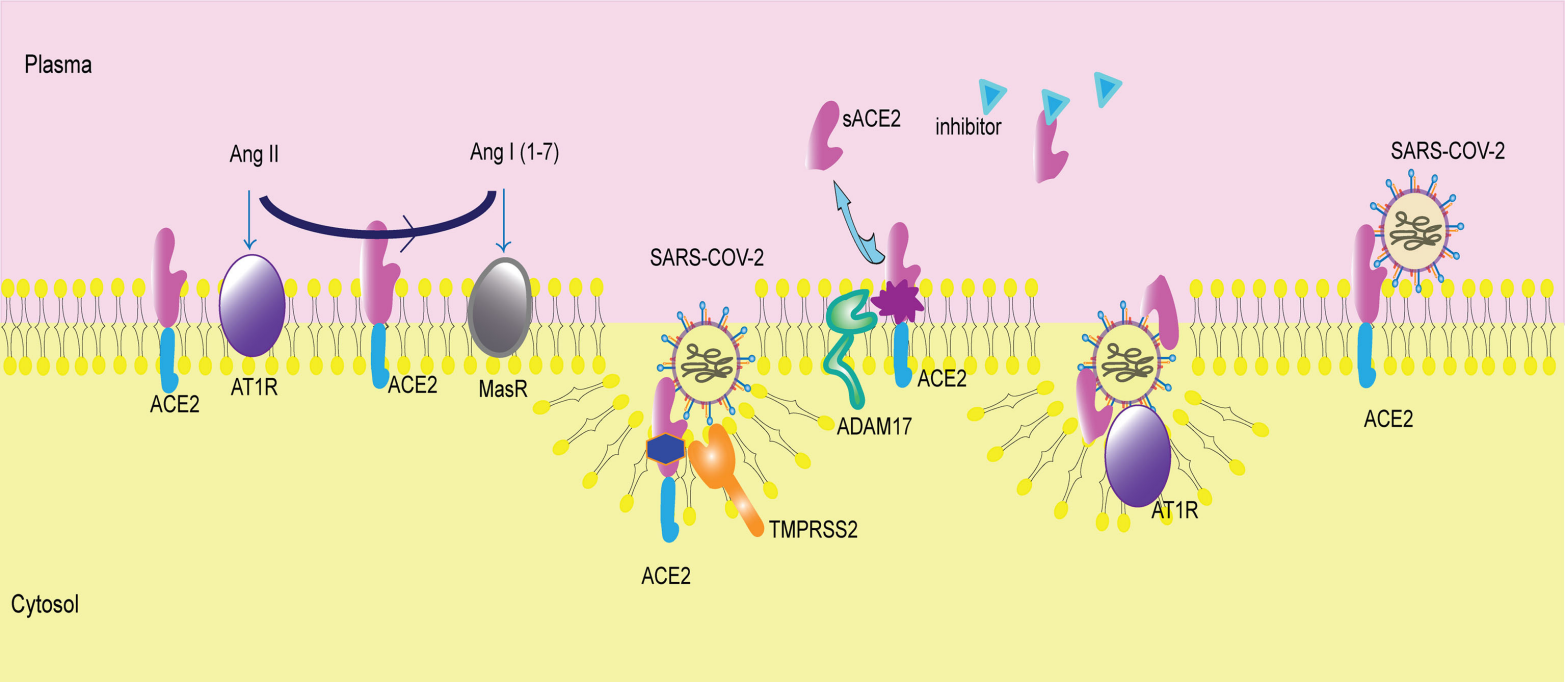
Membrane-bound ACE2 and ACE2 shedding. ACE2 is a membrane receptor. It converts angiotensin II (Ang II) to angiotensin I (1-7) (Ang I(1-7)). Then, Ang II binds to angiotensin type 1 receptor (AT1R), and Ang I(1-7) binds to mitochondrial assembly receptor (MasR). ACE2 is the membrane receptor of SARS-COV-2. Transmembrane protease serine 2 (TMPRSS2) cleaves ACE2 and mediates viral entry into cells. A disintegrin and metallopeptidase domain 17 (ADAM17) catalyzes ACE2 shedding, producing sACE2. The inhibitor of sACE2 is in the plasma, blocking sACE2 activity. sACE2 can bind to SARS-COV-2 and then facilitate virus entry *via* AT1R.

Although sACE2 is believed to catalyze Ang II hydrolysis, an increasing number of circulating ACE2 attenuates its protective role in many tissues and organs and is even detrimental to many organs, such as the heart (Epelman et al., 2008; Jia et al., 2009; Ramchand et al., 2018; Shao et al., 2019). The physiological and pathological effects of sACE2 on specific organs or tissues are exploring, but the potential mechanism was not determined (**Figure 2**).

## sACE2 in COVID-19

### sACE2 Mediates SARS-COV-2 Entry Into Cells

ACE2 has been identified as a SARS-COV-2 receptor on the cell membrane (Hoffmann et al., 2020). What about sACE2, the form that lacks the cytoplasmic region? Recently, two teams demonstrated that sACE2 binds to SARS-COV-2 and then mediates its entry into cells (Karthika et al., 2021; Yeung et al., 2021). These findings suggest a role of ACE2 shedding and sACE2 in SARS-CoV-2 infection. Whether it has a beneficial effect or causes harm, remains to be fully understood.

ACE2 is upregulated 199-fold in cells in bronchoalveolar lavage fluid (BALF) from COVID-19 patients (Garvin et al., 2020). In healthy individuals, circulating ACE2 levels are very low and are difficult to detect (Rice et al., 2006). In COVID-19 patients, sACE2 is significantly elevated in the presence of severe complications or pre-existing cardiorenal conditions (Vassiliou et al., 2021; Lundström et al., 2021; Patel et al., 2021; Kragstrup et al., 2021). In addition, monitoring a critically ill COVID-19 patient revealed that sACE2 dramatically increased at the onset of disease (Nagy et al., 2021). These studies suggest that sACE2 is increased in COVID-19, even correlating with the severity of disease. From our perspective, a higher concentration of sACE2 means a higher binding rate with SARS-COV-2 with increased sACE2-virus complexes. Subsequently, many complexes enter and attack cells and then replicate additional virus, spreading the infection to other sites. On the other hand, the virus-sACE2 complex in the extracellular space can flow toward other areas, causing broad tissue destruction (Rahman et al., 2021).

However, the limitation of the large size of the virus-sACE2 complex may mean that it is unable to cross certain microvessels or spread extensively (Wysocki et al., 2019). ACE2 is expressed by endothelial cells, and the endothelium is considered one of the most damaged areas in COVID-19 (Patel et al., 2016; Amraei and Rahimi, 2020; Varga et al., 2020; Nagy et al., 2021). Thus, we postulated a double-hit theory of SARS-COV-2. Endothelial cells are the first hit target of SARS-COV-2. After the endothelium is damaged, an activated inflammatory response induces a cytokine storm, which is also a lethal reaction to COVID-19. Moreover, the impaired endothelial barrier cannot limit the virus-sACE2 complex from traversing across vessels, allowing the complex to infect additional organs and tissues. This contributes to severe complications such as multiple organ dysfunction syndrome (MODS), which we consider to be the second hit. The double-hit theory may elucidate the elevated level of sACE2 in COVID-19 patients with severe complications.

Notably, the establishment of double-hit theory is based on the occurrence of viremia. SARS-COV-2 was indicated in blood of COVID-19 patients while the sign was associated with the disease severity (Chen et al., 2020; Fajnzylber et al., 2020; Puelles et al., 2020; Tan et al., 2020; Jacobs et al., 2021; Järhult et al., 2021; Li et al., 2021). Critically ill patients were more prone to have viremia than non-ICU patients and outpatients (Tan et al., 2020; Chen et al., 2020; Jacobs et al., 2021). Li et al. illustrated markers corresponding to gastrointestinal tract, liver and pancreas damage increased in viremic individuals (Li et al., 2021). Like IL-6, IL-2, CCL7, CXCL10/IP-10 and other cytokines also elevated in viremic patients (Tan et al., 2020; Li et al., 2021). Although the rational mechanism between MODS, cytokine storm and viremia have not been demystified, these results may explain why plasma viral load correlates to worsen clinical outcome, disease severity and increasing risk of mortality (Tan et al., 2020; Jacobs et al., 2021; Järhult et al., 2021; Li et al., 2021). In addition, the proteomic analysis uncovered that the appearance of viremia was accompanied by sACE2 elevation in blood and endovascular injury, in favor of circulating of the virus-sACE2 complex through the body (Li et al., 2021).

In addition, biomarkers of endothelial injury and inflammation were increased in the advanced stage of COVID-19 and decreased in the convalescence phase. Notably, the increase in E-selectin and IL-6 was parallel, while sACE2 increased following a two-day delay (Tong et al., 2020; Nagy et al., 2021). This suggests that the inflammatory response initiates increased ACE2 shedding with consequently higher levels of sACE2. Along with the double-hit theory, cytokines that induce ACE2 shedding may participate in the spread of virus throughout the body.

SARS-COV and NL63, two human coronaviruses, have been shown to induce ACE2 shedding (Haga et al., 2008; Glowacka et al., 2010). With respect to SARS-COV-2, research on ACE2 shedding is scarce. We speculate that the elevation of sACE2 levels in the plasma is partially attributed to virus-induced shedding. In parallel, a higher concentration of sACE2 is followed by increased virus-sACE2 complexes and more virus production, which forms positive feedback loop. This feedback contributes to the rapid and aggressive nature of SARS-COV-2, lethally invading the body. If this hypothesis is true, regulating ACE2 shedding, such as through ADAM17, may represent a potential therapeutic target.

## sACE2 Inhibition and the Virus

An increasing body of evidence suggests that sACE2 elevation occurs during pathogenesis (Roberts et al., 2013; Ramchand et al., 2018; Ramchand et al., 2020), but it is difficult to assess plasma sACE2 levels in healthy people. One possible reason is that an endogenous inhibitor in healthy human plasma perturbs sACE2 enzymatic activity (Lew et al., 2008). The inhibitor identify and its relationship with activators of ACE2 shedding remain an area of active research. We propose that the inhibitor counterbalances sACE2 activity to maintain homeostasis. Once the concentration of sACE2 becomes sufficiently high, the inhibition is overcome (Lew et al., 2008). The precise concentration of sACE2 at which the inhibition loses efficiency is worthy of investigation. Perhaps the very concentration is the boundary between health and pathology, which could be used in future disease screening. Moreover, sex, geographic ancestry, and BMI are top-level factors determining sACE2 levels (Narula et al., 2020). We need to evaluate and correct for the effects of these factors when determining specific sACE2 levels.

Importantly, existing *in vitro* or organoid experiments ignore that an inhibitor of sACE2 is in human plasma (Lew et al., 2008). Therefore, when the virus enters the human body, will the inhibitor interfere with the interaction between sACE2 and SARS-COV-2? Or can the virus react with the inhibitor? We hypothesize that the inhibitor maintains sACE2 concentrations at physiological ranges due to self-regulation. Perhaps viral infection breaks the balance by initiating more active ACE2 shedding. Given these gaps in knowledge, the network linking sACE2, the inhibitor, and SARS-COV-2 needs to be demystified.

## sACE2 in COVID-19 Treatment

Although the interaction between sACE2 and SARS-COV-2 is not fully understood, current knowledge gives us some inspiration for treatment. Exogenous supplementation with rhACE2 competitively inhibits endogenous sACE2 binding to virus. RhACE2 has already been tested in phase 1 and phase 2 clinical trials (Haschke et al., 2013; Khan et al., 2017). A recent study on COVID-19 illustrated that clinical-grade rhACE2 binding to coronavirus in ﻿engineered human tissues blocks viral infection (Monteil et al., 2020). One case report of a COVID-19 patient observed an improvement in viremia after a 1-day infusion of rhACE2 (Zoufaly et al., 2020).

On the other hand, ACE2 shedding and internalization of the ACE2-virus complex both lead to the loss of membrane-anchoring ACE2, producing increased Ang II and decreased Ang I (1-7). Then, the balance shifts toward the Ang II-AT1R axis, impairing organs and tissues (Gheblawi et al., 2020). Administration of rhACE2 can convert large amounts of Ang II to Ang I (1-7), guiding the Ang I (1-7)-MasR axis and relieving organ injury. In other words, rhACE2 not only binds to the virus but also ameliorates virus-related complications. In response to rhACE2 treatment, one COVID-19 patient with marked enhancement of Ang I (1-7) and decreased Ang II recovered from lung injury, indicating the dual effect of rhACE2 (Zoufaly et al., 2020). This suggests the feasibility and safety of rhACE2 for the clinical treatment of COVID-19. A clinical trial (NCT04335136) with a larger sample size explored the treatment effect of rhACE2 in COVID-19 patients, which ended in December 2020. We hope the fruits of the trial will bring some new ideas.

With the understanding that sACE2 facilitates SARS-COV-2 cellular entry and exacerbates infection, we question whether the interaction between rhACE2 (a type of sACE2) and virus is beneficial. On the other hand, rhACE2 can be synthesized and designed. Elucidating the mechanism of sACE2 in the pathology of COVID-19 helps to produce much safer and more effective therapeutic rhACE2. Recently, researchers engineered sACE2 with three mutations, and the novel decoy receptor was named sACE2_2_.v2.4. These designed mutations lead to sACE2_2_.v2.4 having a higher affinity for SARS-COV-2 than the wild-type ACE2 receptor and the best monoclonal antibody, more potently blocking virus cell entry. In addition, sACE2_2_.v2.4 efficiently neutralized viral infection (Chan et al., 2020; Chan et al., 2021).

Another insightful perspective is that rhACE2 concentrations (~10–200 mg/mL) far beyond the physiological range block SARS-COV-2 infection, while concentrations near the physiological range (i.e., ng/mL level) facilitate virus cell entry (Yeung et al., 2021). This new idea is in line with the idea that sACE2 binding with virus increases its infectivity. Interestingly, the dose of rhACE2 (0.4 mg/kg) and plasma ACE2 (µg/ml) level in COVID-19 patients did not reach the “treatment concentration”, but they did achieve better outcomes (Monteil et al., 2020). The perspective regarding rhACE2 concentration is based on an *in vitro* cell model (Yeung et al., 2021). In our view, after rhACE2 enters the body, the subsequent reaction between the virus or other factors remains mysterious, which may contribute to the discrepancies in findings. It is difficult to determine the pharmacokinetics and pharmacodynamics of rhACE2, as we cannot distinguish it from endogenous sACE2. Therefore, we are unsure whether rhACE2 binding to SARS-COV-2 interferes with both the detection of rhACE2 and the protective effect. Therefore, defining the concentration of rhACE2 appropriate for COVID-19 treatment or other clinical practice is vital and warrants deeper study.

## Discussion

A topic of interest includes membrane-bound ACE2 being the receptor of SARS-COV-2. However, the shedding process and soluble form of ACE2 are active considerations. In this minireview, we focused on the role of sACE2 in COVID-19 and the therapeutic use of rhACE2 in COVID-19. In line with ACE2, sACE2 can bind to SARS-COV-2, mediating virus entry into cells. In an investigation of COVID-19 patients, sACE2 levels were increased in BALF and serum. Furthermore, levels of sACE2 are positively correlated with disease severity. Based on this observation, we propose a double-hit hypothesis to explain the pathological progress of COVID-19 and emphasize endothelial injury at the onset of COVID-19. Additionally, inflammation may participate in ACE2 shedding, worsening COVID-19-related complications. Understanding the underlying mechanism between sACE2 and virus enlightens therapy for COVID-19. Infusion of rhACE2 exogenously replenishes ACE2, prevents organ injury and potentially improves clinical symptoms. The specific efficacy of rhACE2 in COVID-19 patients is currently undergoing clinical trials. Certainly, the effective dose of rhACE2 for treatment is controversial and warrants careful investigation.

## Author Contributions

JW, HZ, and YA contributed to the conception of the manuscript. JW and HZ searched the literature and drafted the manuscript. The authors read and approved the final manuscript.

## Funding

The work was supported by Beijing Municipal Natural Science Foundation, China (Grant No. 7212124). HZ was supported partially by research fund provided by Peking University People’s Hospital Research and Development Funds (No. RDY2019-43, derive sepsis phenotypes using electronic medical data and machine learning).

## Conflict of Interest

The authors declare that the research was conducted in the absence of any commercial or financial relationships that could be construed as a potential conflict of interest.

## Publisher’s Note

All claims expressed in this article are solely those of the authors and do not necessarily represent those of their affiliated organizations, or those of the publisher, the editors and the reviewers. Any product that may be evaluated in this article, or claim that may be made by its manufacturer, is not guaranteed or endorsed by the publisher.

## Abbreviations

ACE2, Angiotensin Converting Enzyme 2; ADAM17, A Disintegrin and Metallopeptidase Domain 17; Ang I, Angiotensin I; Ang I (1-9), Angiotensin I (1-9); Ang II, Angiotensin II; Ang I (1-7), Angiotensin I (1-7); AT1R, Angiotensin II Type 1 Receptor; BALF, bronchoalveolar lavage fluid; CLD, collectrin-like domain; COVID-19, Coronavirus disease 2019; MasR, Mitochondrial assembly receptor; MODS, multiple organ dysfunction syndrome; PD, protease domain; PMA, phorbol ester; RAS, Renin-Angiotensin-System; RBD, receptor binding domain; rhACE2, recombinant human angiotensin converting enzyme 2; sACE2, soluble angiotensin converting enzyme 2; SARS-COV-2, severe acute syndrome coronavirus; Sema4D, Semaphorin 4; TACE, tumor necrosis factor α-converting enzyme; TMPRSS2, transmembrane protease serine 2.
